# An asymmetry in wave scaling drives outsized quantities of coastal wetland erosion

**DOI:** 10.1126/sciadv.adj2602

**Published:** 2023-11-08

**Authors:** Rusty A. Feagin, Kuang-An Chang, Thomas P. Huff, Ignacio Rodriguez-Iturbe, Jin-Young Kim, James Kaihatu, Nicoletta Leonardi, Sergio Fagherazzi

**Affiliations:** ^1^ School of Geography and the Environment, University of Oxford, Oxford, UK.; ^2^ Department of Ecology and Conservation Biology, Texas A&M University, College Station, TX USA.; ^3^ Department of Ocean Engineering, Texas A&M University, College Station, TX, USA.; ^4^ Department of Civil and Environmental Engineering, Texas A&M University, College Station, TX, USA.; ^5^ Engineering and Research Development Center, US Army Corps of Engineers, Vicksburg, MS, USA.; ^6^ University of Texas, Arlington, Arlington, TX, USA.; ^7^ Department of Geography and Planning, University of Liverpool, Liverpool, UK.; ^8^ Department of Earth and Environment, Boston University, Boston, MA, USA.

## Abstract

Wetland shorelines around the world are susceptible to wave erosion. Previous work has suggested that the lateral erosion rate of their cliff-like edges can be predicted as a function of intercepting waves, and yet numerous field studies have shown that other factors, for example, tidal currents or mass wasting of differing soil types, induce a wide range of variability. Our objective was to isolate the unique effects of wave heights, wavelengths, and water depths on lateral erosion rates and then synthesize a mechanistic understanding that can be applied globally. We found a potentially universal relationship, where the lateral erosion rates increase exponentially as waves increase in height but decrease exponentially as waves become longer in length. These findings suggest that wetlands and other sheltered coastlines likely experience outsized quantities of erosion, as compared to oceanic-facing coastlines.

## Teaser

A key asymmetric relationship in wave scaling was discovered to drive outsized quantities of lateral wetland erosion.

## INTRODUCTION

Waves erode wetland shorelines in a wide variety of locations around the world (*1*). An important and open question is whether the lateral erosion of their edges, or *E*, can be predicted as a function of the intercepting waves. As a first guess, early work (*2*) posited that the energy and erosive shear stress generated by a single wave traveling through deep water should be roughly proportional to the square of its wave height, or *H*^2^ (a list of all variables can be found in Materials and Methods). Taking this line of thinking a step further, analytical (*3*) and numerical (*4*) works have suggested that the lateral erosion of a vertical wetland edge is linearly related to the wave power, or a combination of *H*^2^, and the wave period *T* where *E* ∝ (*H*^2^*T*)*^x^* with *x* = 1.

In contrast, however, a wide range of field-based studies have identified a nonlinear fit between lateral erosion and wave power, with *x* = 1.10 to 1.37, e.g., (*5*–*10*). These studies have shown a large amount of variability in erosion across field sites, caused by differences in soil properties, vegetation, and myriad other factors (*11*). While these inherent differences among the sites can be partially accounted for by standardizing the individual erosion measurements by the mean quantity of erosion at each site (*12*), nonwave erosive effects still contaminate the field datasets and raise the exponent *x*. For example, mass wasting driven by gravity (*13*), tidal creek flows (*14*–*15*), alongshore current velocity–driven erosion (*11*, *16*), precipitation-driven erosion (*17*), and soil cracking due to wetting and drying effects (*18*) each occur at different frequencies across time (*19*–*23*).

A longer duration field study generally will have (i) more of these nonwave effects embedded within the reported measurements as the duration of the study increases and (ii) a higher average wave power as the likelihood of encountering large wave events increases over time—with the net effect raising the exponent. These nonwave effects also occur in variable quantities depending on each unique study, which further induces scatter into the generalized nonlinear fit, particularly as the wave power increases (*11*). Both inherent site variation and these nonwave effects have complicated the discovery of a more universal relationship between lateral erosion and wave mechanics.

Our overall objective was to identify the relationship between lateral erosion and several key wave parameters, after isolating these extraneous effects. To do so, we first collected several empirical datasets in the laboratory to explore the relationship between lateral erosion, *E*, at the shoreward position of a vertical edge of wetland, and wave height *H*, wavelength *L*, and water depth $h_{*}$ (see Materials and Methods below). Our laboratory datasets isolated the unique effect of each of these parameters to wetland edge erosion, including the variability in the vertical dimension, while excerpting the nonwave erosive effects that accumulate over time (our experiments measured wave erosion on the order of seconds at a maximum duration of 30 min per trial). From among many convolutions of the variables, we then found the best fit with *E* and sought to describe the physics of the wave conversion–to–erosion process from a first-principles perspective.

In this same laboratory dataset, we next explored the statistical properties of the individual lateral erosional depths, or “chunks” with a depth of *e*, that occurred across the vertical face of the eroding edge and related them back to *E*. We then collected lateral erosion data from across three continents at field sites with a variety of *H*, *L*, and $h_{*}$ conditions, as documented further in (*12*). We mean-standardized the left and right sides of Eq. 1 by site location that converted them into what (*12*) refers to as E* and P*, respectively, and inserted the laboratory data (see Materials and Methods below).

Our results led us toward a contemporary theoretical conception of how waves induce lateral erosion, showing that the erosive effect of a wave’s height versus its length is dependent on the slope of the eroding edge. In the case of vertically oriented edges along wetland shorelines, lateral erosion rates increase exponentially as waves increase in height but decrease exponentially as waves become longer in length.

## RESULTS

### Edge erosion in the laboratory

With our laboratory work, we first found that lateral erosion *E* was a nonlinear function of wave power as one might expect when holding all other variables constant, similar to the aforementioned field studies, although with a far lower exponent of *x* = 0.65 (figs. S1 and S2). We attribute this lower value to the removal of nonwave effects, by working in the laboratory and over relatively short time scales.

**Fig. 1.**
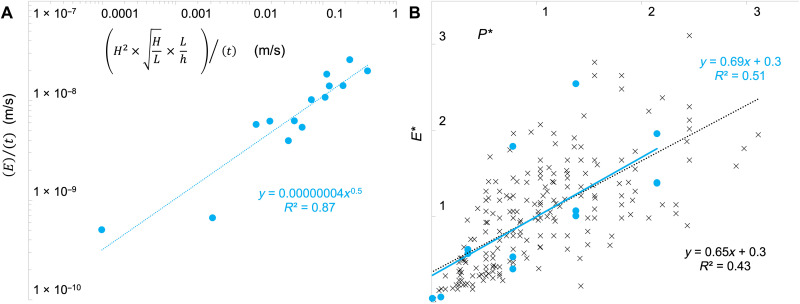
The laboratory and field data exhibited a similar fit between the wave parameters and lateral erosion, once the nonwave factors were standardized. (**A**) The relation $E\propto {\left(H^{2}\times \sqrt{\frac{H}{L}}\times \frac{L}{h_{*}}\right)}^{\zeta }$ with ζ = 0.5 provided the best fit for laboratory flume data sets. Note that, here, ζ = 0.5 is embedded in the regression fit as opposed to listed on the *x* axis; dimensionless erosion can be obtained by stacking the two sides; in this depiction, the units for both axes were divided by time duration *t* and thus were in meters per second empirically. (**B**) The field site data from across three continents (black markers) exhibited a similar but more scattered relationship than the laboratory data (blue circles), when both were graphed as mean-standardized erosion E* versus wave power P*. Units for both axes are dimensionless.

However, we found that the best fit was more complex and included a permutation of multiple variables (Fig. 1A). While increasing the wave height *H* generally increased the cross-shore orbital velocities *u* in the water column, the water depth at the immediate edge $h_{*}$ also affected them by altering the breaking wave form and thus the vertical breadth, location, and magnitude of erosion (figs. S3 and S4). The best statistical fit for the data included the wave height, steepness, and the breaking form effects as

$$E\propto {\left(H^{2}\times \sqrt{\frac{H}{L}}\times \frac{L}{h_{*}}\right)}^{\zeta }$$ (1)

with ζ = 0.5. In this formulation, the lateral erosion was proportional to the product of the wave energy, the square root of the wave steepness, and the inverse of the relative water depth (which accounted for breaking form effects). Both sides of the equation could also be divided by time duration *t* to obtain a lateral erosion rate (as depicted in Fig. 1A). The scaling exponent ζ described the transformation from two-dimensional wave geometry into one-dimensional lateral erosion (see Supplementary Equations and Text).

**Fig. 2.**
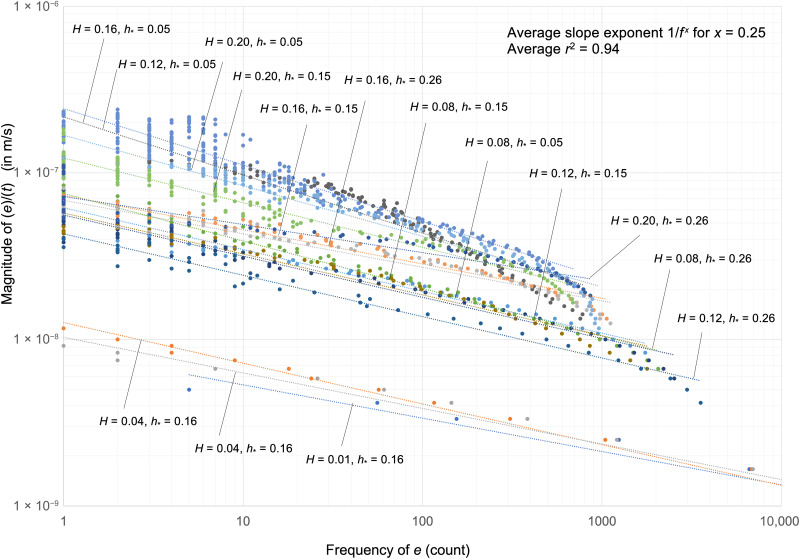
The magnitude of individual erosion depths, e, was inversely related to their frequency of occurrence, f, over a wide range of scales. Each dot represents the number of times that a given depth was eroded on the vertical surface of the wetland edge, during the induced *H*/$h_{*}$ wave conditions in the laboratory (each of these conditions is denoted by the colored linear regression fit lines). The data are depicted as erosion per unit time *t*, in meters per second, similar to Fig. 1A). The exponential fit for the slope was *e* = 1/*f*^0.25^ with *r*^2^ = 0.94, when averaged across all wave height *H*/$h_{*}$ combinations.

For this same laboratory data, *E* was in fact the mean of a large number of individual lateral erosional depths *e* that occurred across the vertical face of the eroding edge (fig. S5). We found that the spatial frequency *f* of encountering these *e* values was inversely proportional to their magnitude, over a wide range of spatial scales (Fig. 2).

Relatively larger *e* depths were fewer in number, and this limitation exhibited reasonably consistent power law behavior over a range of frequency scales where *e* = 1/*f*^0.25^. This range for *e* was bounded on its lower magnitude end by the mean of the distribution, which was equivalent to *E* (figs. S6 and S7). The distribution was long-tailed and positively skewed toward larger *e*. One potential explanation for this distribution was that the dynamics of the mechanical erosion process were dampened, with negative feedback limiting the number of the deeply eroding chunks of material. This behavior consistently scaled across all *H*.

### Edge erosion in the field

We found nearly the same statistical fit across the field sites for E* (Fig. 1B) as we had found for the laboratory data for *E*, suggesting that Eq. 1 provided at least a first-order approximation of the relevant dynamics across the sampled field locations. The field data showed greater scatter and a lower *r*^2^ than the laboratory data, as expected from data distributed across many site locations around the globe. However, because E* had been standardized by site location, the remaining variance was less likely to be due to differences in soil erodibility caused by soil properties or vegetation. Rather, this variance was likely due to nonwave processes such as mass wasting and slumping that acted differentially over the unique time lengths for which each data point had been collected or a complex combination of these factors.

To further highlight this concept, we converted Eq. 1 into units of work that accounted for cross-site variations in soil erodibility. For consistent units of measurement in the equation, we first squared both sides, and then, on the right-hand side, inserted the common terms for wave energy density $\frac{1}{8}\rho gH^{2}$ in place of *H*^2^ and the shallow water wave period $T\sqrt{gh}$ in place of *L* (or alternately, the deepwater wave period $\frac{g}{2\pi }T^{2}$; see Supplementary Equations and Text). On the left-hand side, we added the bulk density of the sediment ϕ and gravitational acceleration *g* of its movement. We then redistributed and separated out the constant terms, yielding

$$E^{2}\varphi g=H^{2.5}\times T^{0.5}\times \frac{\rho g^{1.25}}{8{h_{*}}^{0.75}}$$ (2)

where both sides were made into units of work (in kilograms meter-squared per second-squared, or joules) through selective cancelation or otherwise dimensionless through stacking (see Supplementary Equations and Text).

Thus, on the basis of the bulk density, other sediment, or site erosion characteristics (in particular, ϕ on the left hand side of Eq. 2), the data points in Fig. 1A could be shifted vertically along the *y* axis, while the relative slope of the regression line remained the same, as a function of the wave conditions (right hand side); this was the reason behind the common slope across site location in Fig. 1B, upon mean standardization. That is, site locations with different soil erodibilities had different absolute erosion quantities, but the relative scaling of the lateral erosion as a function of the wave conditions was constant across sites.

## DISCUSSION

### Asymmetry between the individual components of wave power and edge erosion

The laboratory data isolated the contribution of *H*, *L*, and $h_{*}$ to lateral erosion, as individual factors in an empirical manner and with variability in the vertical dimension. Although there have been many past studies that have investigated the effects of waves on wetland erosion, these studies were conducted over longer time periods in the field, and, thus, their measured erosion rates also reflected the influence of nonwave erosion processes. In the existing laboratory studies that did exist, e.g., (*18*), the purpose was to induce these nonwave processes. We controlled the laboratory conditions to exclusively investigate the continuous impact of waves alone over relatively short time scales (seconds to minutes).

Using this laboratory dataset, an asymmetric relationship between lateral erosion and the two components of wave power, *H* and *T*, emerged from Eqs. 1 and 2 when using shallow water formula and removing the constants and depth as

$$E\propto H^{1.25}T^{0.25}$$ (3)

or alternately as *E* ∝ (*H*^2.5^*T*^0.5^)^ζ^ with ζ = 0.5. Equation 3 provides the best and most simple fit for the controlled, wave-only conditions (figs. S1 and S2).

As the exponents in Eq. 3 explicitly describe, lateral erosion is more efficient when the wave power is packed into the wave height as opposed to length. That is, lateral erosion rates nonlinearly increase as waves increase in height but nonlinearly decrease as waves become longer in length.

### Eroding surface geometry as a mechanism for the asymmetry

We can potentially explain the mechanism that caused the asymmetry by generating a hypothesis that states that the geometry of the individual erosion depths *e* differentially limits the erosive effect of increasing the wave height *H* versus the wavelength *L*. The following description not only used our knowledge of the empirical 1/*f*^0.25^ distribution of the *e* erosional depths but also greatly simplified the relevant processes by assuming that the waves were sinusoidal and that their relative water velocities were distributed by distance to the mean water line (see Supplementary Equations and Text). An expansion to nonlinear waves would be straightforward mathematically (it would only slightly alter the relations, while achieving the same general effect as our wave steepness parameter $\sqrt{H/L}$ in Eq. 1, but much more difficult to denote and conceptualize). However, we need only a first-order description to identify the relevant geometry at play.

**Fig. 3.**
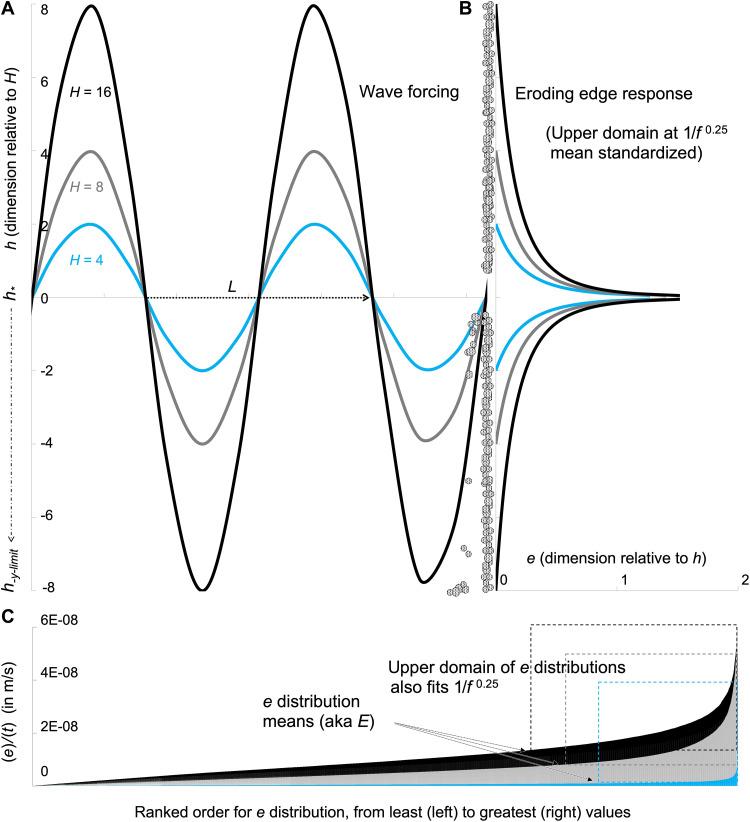
A conceptual depiction of the simplified, mechanistic hypothesis, depicting the relative geometry of the waves and the eroding edge. Nonlinear, breaking, and *H*/*h* transformed waveforms are not depicted for ease of interpretation of the primary relationship. (**A**) As waves of varying height *H*, but constant *L*, strike a hypothetical edge, (**B**) their horizontal erosion depths *e* are proportional to position along a vertical *h* dimension following a 1/*f*^0.25^ power law. (**C**) This power law describes the behavior of the upper domain of the *e* distribution, when it is standardized by the distribution mean, or *E*. Part (A) and (B) depict the proportional relations only between *H*, *L*, and *e*; they are unitless as depicted. Part (C) depicts empirical laboratory data ranked in order of *e* (per time *t*, in meters per second), using *H* = 16 with *h* = 0.15 (black), *H* = 8 with *h* = 0.15 (gray), and *H* = 4 with *h* = 0.16 (blue) as examples. The upper domain of these empirical distributions fit the power law (see text, Fig. 2, and figs. S8 and S9).

First, we set a generalized vertical dimension *h* to record all possible water levels that intercepted the edge during a *H* wave and recorded them as distances *h_y_* from the mean water depth $h_{*}$ (Fig. 3A). The maximum and minimum of this wave reached *h*_±*y*−limit_ on either side of $h_{*}$, which then set the wave height along this dimension as *H* = 2(*h*_±*y*−limit_). With no alongshore variation in the laboratory waves, we assumed that the variation in *e* was due to this vertical dimension alone and that the erosion was greatest at $h_{*}$. Using the known frequency distribution (Fig. 2), we accordingly made each *e* proportional to its vertical distance from $h_{*}$ in the *y* direction (Fig. 3B), writing this as *e_y_* ∝ (1/*h_y_*)^0.25^.

Next, we built a relation between the one-dimensional (horizontal erosional depth) measure of *E* and the sum of all one-dimensional *e_y_* erosion depths across *h_y_*, as $E\propto 2\int_{h_{*}}^{h_{\pm y-\mathrm{limit}}}e_{y}(h_{y})$ and thus $E\propto 2\int_{h_{*}}^{h_{\pm y-\mathrm{limit}}}{\left(1/h_{y}\right)}^{0.25}(h_{y})$. Its right-hand side then became 2(*h*_±*y*−limit_)^1.25^ after integrating *e* across all *h_y_*. With substitution of *H* for 2(*h*_±*y*−limit_) as described in the paragraph above, the erosion rate then scaled as *E* ∝ *H*^1.25^. This rate was equivalent to *E* ∝ (*H*^2.5^)^ζ^ with ζ = 0.5, as one could find from the wave height terms alone in Eqs. 1 and 3.

The *e_y_* ∝ (1/*h_y_*)^0.25^ proportionality occurred within a domain of the *h_y_* dimension that stretched from the largest *e* located at the central portion of the edge at $h_{*}$, out to the mean *e*. For *e* smaller than the mean (out toward the upper and lower portion of the eroding edge), we wrote *e_y_* ∝ (1/*h_y_*)^1^ because the proportionality scaled linearly in this second domain empirically (figs. S8 and S9). The additive result of the two domains for the entire distribution was trivial as the inner domain ultimately drove the asymmetric scaling relation of *E* ∝ *H*^1.25^.

The result of *E* ∝ *H*^1.25^ was that as waves increase in height alone, their ability to cause erosion became increasingly more efficient because of the geometry of the eroding surface. The erosion rate increased nonlinearly as wave heights became larger. The laboratory data and the statistical geometry of *e* depths exhibited this scaling relationship empirically, and our hypothesis provided a potential mechanistic explanation.

Within our datasets, the steep erosional pockets composed of *e* depths became increasingly deep at an exponential rate in statistical terms. We suggest that a wave’s penetration efficiency into these pockets was likely limited by negative feedback with the internal angle of the eroding surface, and an increase in wave height better exploited the geometry of the total edge by biasing more of its energy to areas outside of these pockets. However, we note that the spatial patterning was somewhat more heterogeneous across *h* than in our hypothesis, although it still appears to be a statistically valid explanation (e.g., Fig. 3B versus fig. S4). Conversely, an increase in the wavelength likely put the wave energy into the horizontal dimension, which was more easily reduced by these pockets through friction induced into the water column (see Supplementary Equations and Text). Although we describe the statistical mechanics and their relation to our empirical datasets herein, there will need to be more future work done to address the wave penetration physics in greater detail.

### Eroding surface slope as a precondition of the asymmetry

The key inclusion of the wave height–to–wavelength ratio term in Eq. 1 (i.e., $\sqrt{H/L}$) and through conversion into *T* for Eqs. 2 and 3 transformed the wave power into appropriately scaled *h* versus *e* erosion terms. Notably, the Iribarren number ξ is known to reduce to $\sqrt{L/H}$ at 45° [i.e., (*24*)], and this fact reinforces the idea that the *h* versus *e* erosional shape is not only likely dependent but also universally relatable through the slope of the erosional surface.

Once an erosional surface tilts toward a 45° angle, then the erosional height to length process likely equilibrates such that the differential becomes irrelevant, *e_y_* ∝ (1/*h_y_*)^1^, and the erosion will scale linearly with the wave power, as dimensional analysis suggests (i.e., *E* ∝ *H*^2^*T*). A dimensional analysis for wetland edges (*3*) thus could be revised to require symmetry between *e* and *h* as a precondition, implying that there is no extra cost to eroding more deeply into narrow spaces, as opposed to the open face of a surface. Once the slope decreases below 45° and toward horizontal, increasing *L* could become a more efficient mode of increasing erosion (i.e., *E* ∝ *H*^0.25^*T*^1.25^).

Differential and asymmetric scaling in erosion should be expected when stretching a wave in only one direction and impacting a non-45° surface, but it has not been considered by previous work on lateral wetland erosion. Further investigations will be required to more fully explore the relevance of this concept across a wide range of coastal slopes.

### Effects of the asymmetry on coastal wetland erosion

Real-world outcomes depend on these fundamentals, regarding the effect of wave heights, lengths, and water depths on wetland erosion. While water waves propagate across more than 73% of Earth’s surface, the relative proximity of the coastline to the location of wave genesis greatly affects the wave height–to–wavelength ratio (i.e., $\sqrt{H/L}$). In general, the waves that strike wetland edges cross-relatively short fetch distances and were created by local wind events. These types of waves are short in wavelength (typically on the order of ~1 cm to 1 m) and steep. In contrast, the waves that strike open ocean shorelines are relatively long in wavelength (~1 to 10 m), with the wave spectrum having been more strongly organized via wave dispersion. That is, given the same wave height, a wave generated in a small water body will be more likely to be steeper (because of shorter wavelength).

As a direct consequence of the asymmetry and as shown by our results, the erosion rate will be generally higher in smaller water bodies for waves of a given height, with all other parameters being equal. Wetland edges should be particularly affected by the asymmetry in an out-sized manner, as they are located in relatively sheltered locations.

Similarly, we should also expect that erosion has increased more rapidly on vertical edge surfaces as compared to surfaces less than or equal to 45°, such as on sandy beaches. We can take the concept even a step further, for example, given that significant wave heights have increased 7% across coastal portions of the globe from 1986 to 2005 due to wind increases (*25*). As a consequence of the nonlinear relationship between wave height and erosion, we might expect to find 18% more erosion at the end of this time period. Many fresh hypotheses, derivative from the asymmetric relationship, can now be tested with existing data, models, and global imagery of shorelines. These erosional outcomes may be unexpected, yet we contend that they will be a direct outcome of the asymmetry.

We note that our findings are specific to the processes of wave erosion only. By design, our approach did include the nonwave effects that can also be related to wetland edge erosion. Critically, these nonwave effects may differentially affect macrotidal, mesotidal, and microtidal wetland edges. For example, there is likely a greater quantity of soil erosion for taller macrotidal edges that is driven by slumping or cantilever failure, and this erosion could affect the scaling rate of erosion and induce scatter into field datasets when lumped across edge height and tidal range. However, for the wave processes alone, our equations removed this effect due to the relative scaling of *H* versus *h* within them. Moreover, other nonwave processes, such as tidal creek flows, alongshore current velocity–driven erosion, precipitation-driven erosion, and soil cracking due to wetting and drying, these effects were similarly removed by our laboratory setup. The purpose of our study was to remove these extraneous effects and focus solely on the waves.

In summary, as waves increase in height and affect a vertical surface, the lateral erosion process becomes more efficient, yet, simultaneously, as waves increase in length, the process becomes less efficient. This seeming contradiction is explained by asymmetric scaling between the individual components of the wave power (wave height versus wavelength) and lateral erosion of a vertical edge. The first-principles formulation, the scaling relation, and empirical laboratory and field results thus point to a contemporary way of conceiving of wetland erosion on vertical edges. The identification and use of a physical description of this process will help scientists, managers, and policymakers better mitigate the loss of shorelines around the world.

## MATERIALS AND METHODS

### Laboratory

We extracted 0.6 m by 0.3 m (horizontal footprint) by 0.15 m (vertical) wetland edge samples and then placed them into a 22.9 m by 36.6 m by 1.2 m wave basin [see movie S1; see (*9*, *26*) for more details]. We subjected them to a range of regular wave heights (0.01 to 0.16 m), water depths (0.05 to 0.26 m), and wave lengths (1.44 to 3.20 m) common to eroding edges. Water depths were measured relative to the bottom of the samples. Samples were collected from along the immediate edge of natural marshes in West Galveston Bay, Texas, and prepared using established methods to ensure minimal compaction and a consistent incident surface [e.g., (*27*)]. We recorded the lateral erosion using a terrestrial laser scanner light detection and ranging sensor (LIDAR) and an ultrasonic sensor at 0.5-mm resolution and then extracted the vegetative roots from the point clouds manually [see (*28*)]. The lateral distance between the before versus after point locations was recorded as an individual erosion depth measurement, *e*. For each wave height by water depth by wavelength trial, we found the average across all *e* measurements and then divided it by the time duration of the trial, to find the lateral erosion *E* per time *t*. The duration of the trials were on the order of minutes to remove nonwave effects (longest was 30 min).

### Field and literature

Using data from across three continents, we first graphed dimensionless erosion E* and dimensionless wave power P* for the summary dataset of Leonardi *et al.* (*12*). This dataset included site-standardized, original field data collected in (*2*, *5*, *29*–*32*). We added four data points that we collected from a field site in West Galveston Bay, Texas (same location where the laboratory samples had also been collected); These data were collected over 318 days along a 20-m-long eroding marsh edge of 0.6 m in height, using the terrestrial laser scanner LIDAR and an ultrasonic sensor to measure the wave parameters, as described in (*28*, *33*). For our laboratory values from Fig. 1A, we first calculated *P* using the equation of Leonardi *et al.* (*12*) (*P* = *Wc_g_*, where *W* = ρ*gH*^2^/8 and *c_g_* is the group velocity which is equivalent to *L*/*T* for the regular laboratory waves). We then followed their method of mean standardization to find P* and E* for each record.

### Variables used in equations

*E* is the lateral erosion in the shoreward direction, mean (in meters); *H* is the wave height (in meters); *L* is the wave length (in meters); $h_{*}$ is the water depth at the erosional edge (in meters); ζ is the transfer efficiency (unitless); *t* is the time duration (in seconds); *u* is the water velocity in the cross-shore dimension (in meters per second); *e* is the lateral erosion of edge, specific to vertical or horizontal position (in meters); *f* is the frequency of occurrence for *e* (count); *h*, *h_y_*, and *h*_±*y*−limit_ are the vertical dimensions of eroding edge, distance from $h_{*}$ on *y* axis (in meters); *k* is the wave number (per meter); ω is the angular frequency (per second); E* is the lateral erosion, standardized by mean *E* from across-site data (unitless); P* is the lateral erosion, standardized by mean *P* from across-site data (unitless); *T* is the wave period (in seconds); ρ is the water density (in kilograms per meter); *g* is the gravitational acceleration (in meters per square second); ϕ is the sediment density (in kilograms per cubic meter); and ξ is the Iribarren number (unitless)

## Supplementary Materials

### This PDF file includes:

Figs. S1 to S9 Legend for movie S1 Supplementary Equations and Text
