# Supplementary material for: An asymmetry in wave scaling drives outsized quantities of coastal wetland erosion
Source: Sci Adv. 2023 Nov 8;9(45):eadj2602. doi: 10.1126/sciadv.adj2602 (PMC10631725; doi:10.1126/sciadv.adj2602)
Supplement: Supplementary file 1 — Figs. S1 to S9 Legend for movie S1 Supplementary Equations and Text [file sciadv.adj2602_sm.pdf]

Science Advances
AAAS

Supplementary Materials for

An asymmetry in wave scaling drives outsized quantities of coastal wetland erosion

Rusty A. Feagin *et al*.

Corresponding author: Rusty A. Feagin, feaginr@tamu.edu

Sci. Adv. **9**, eadj2602 (2023)
DOI: 10.1126/sciadv.adj2602

The PDF file includes:

Figs. S1 to S9
Legend for movie S1
Supplementary Equations and Text

Other Supplementary Material for this manuscript includes the following:

Movie S1

Fig. S1. Lateral erosion plotted against the traditional definition of wave power, where the exponent fits as x = 0.65. Only laboratory data is included.

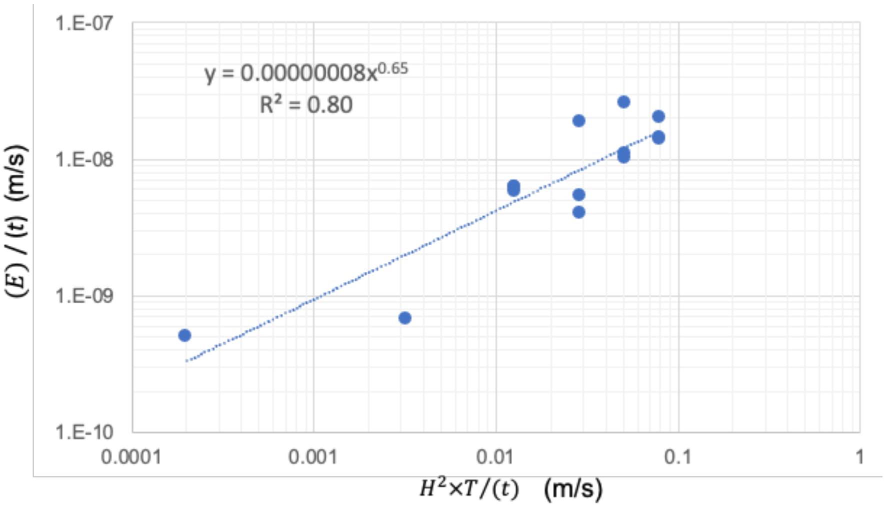

Fig. S2. Lateral erosion plotted against asymmetric wave power, where the exponent fits as x = 1. Only laboratory data is included. The fit is linear with an exponent of 1 – thus the "wave power" scales asymmetrically with respect to lateral erosion as $H^{1.25}T^{0.25}$, when isolating the wave processes and excerpting mass wasting or any other processes. Still, the full version of Eq. 1 has a better r^2 fit and is dimensionless when stacked (as opposed to the simplified Eq. 3 wave power relationship displayed here) – this is because Eq. 1 also includes the relative water depth, and is thus more universal.

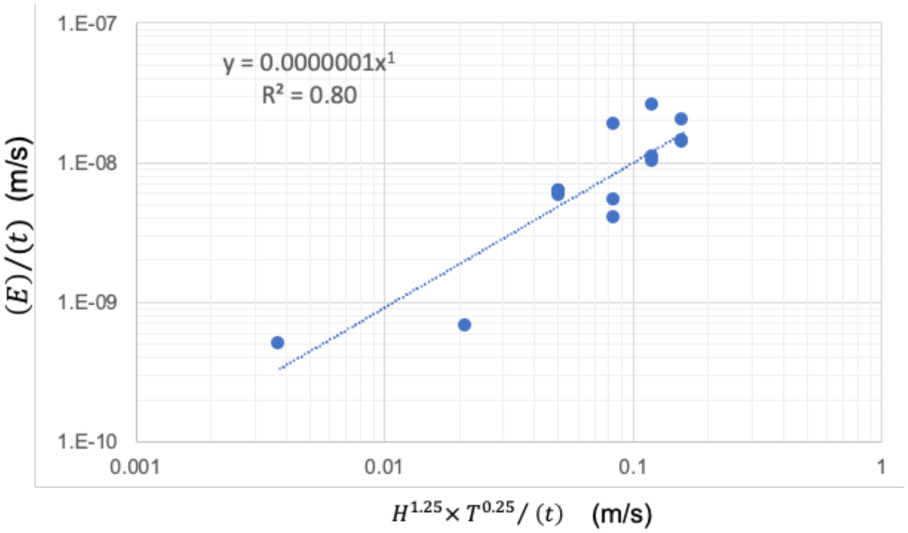

Fig. S3. $\boldsymbol{H^2 \times \sqrt{\frac{H}{L}} \times \frac{L}{h}}$ **versus incident water velocity** $\boldsymbol{u}$ **(m/s), as recorded in the laboratory.** Water velocity is in the cross-shore dimension (i.e., intercepting the marsh edge from a horizontal direction). Assuming the fit of $H^2 \times \sqrt{\frac{H}{L}} \times \frac{1}{h}^{\zeta}$ with $\zeta = 0.5$ over a given duration of time, e.g. 1 sec, yields the relationship dimensionless.

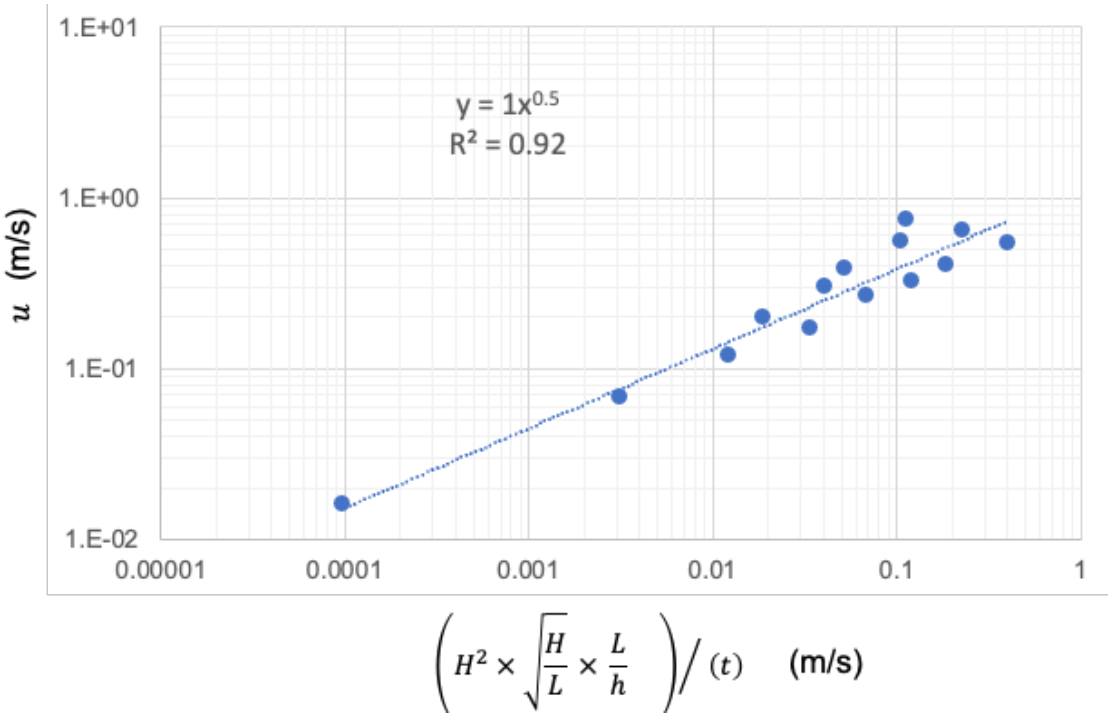

Fig. S4. Erosion depths *e* varied vertically across the *h* dimension of the marsh edge. Erosion depths for various wave heights H (as depicted by the colored lines) when the still water level h_* (as depicted by the dotted line) was (A) near the top, (B) at the mid-point, and (C) near the bottom of the wetland edge.

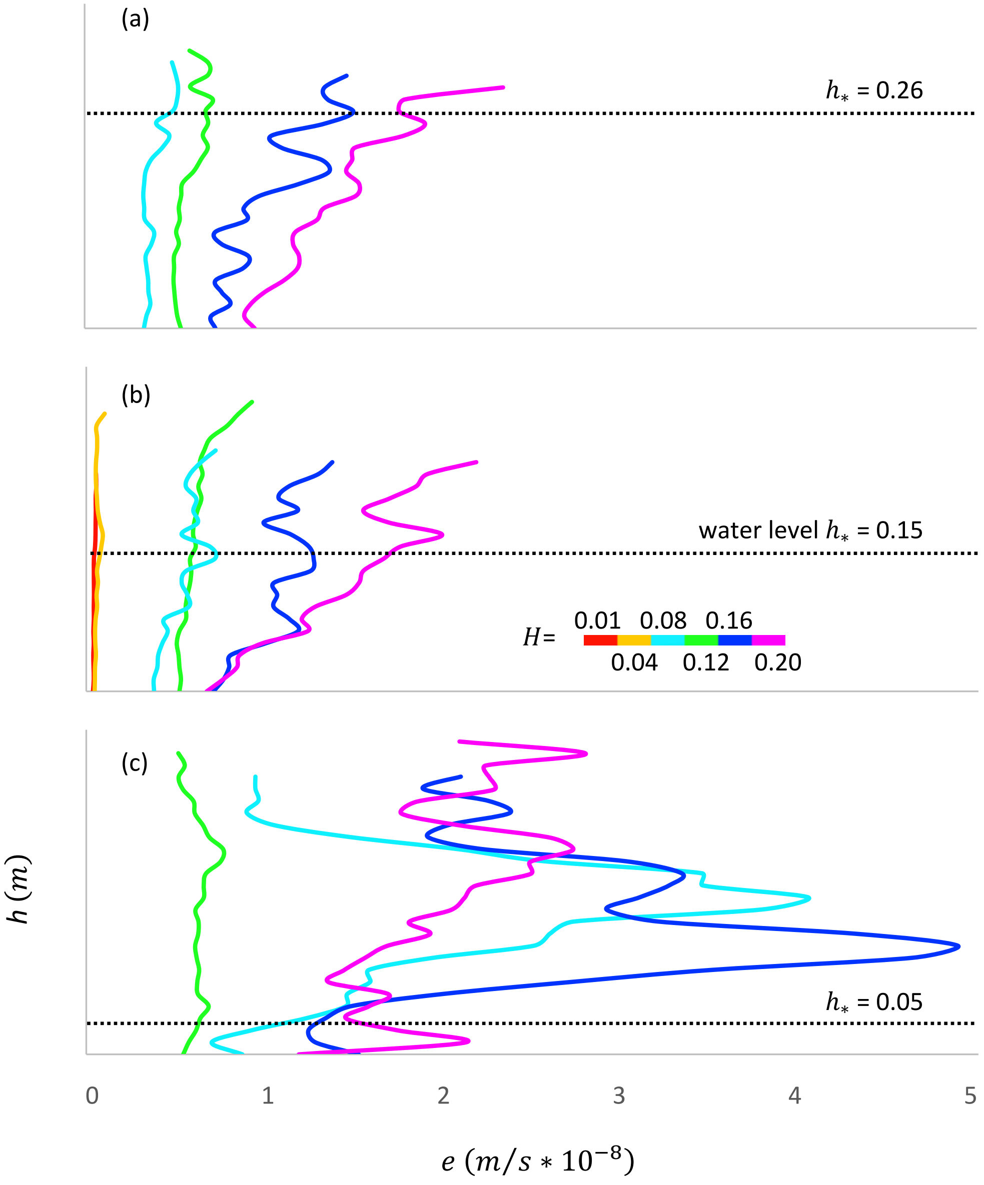

Fig. S5. The cross-sectional area of erosion plotted against the various relations described in the main text. Again, $\zeta = 0.5$ provided the best linear fit. In this conception, the sum of all e depths is depicted across the vertical dimension h, similar to that depicted in Fig. 3 of the main text.

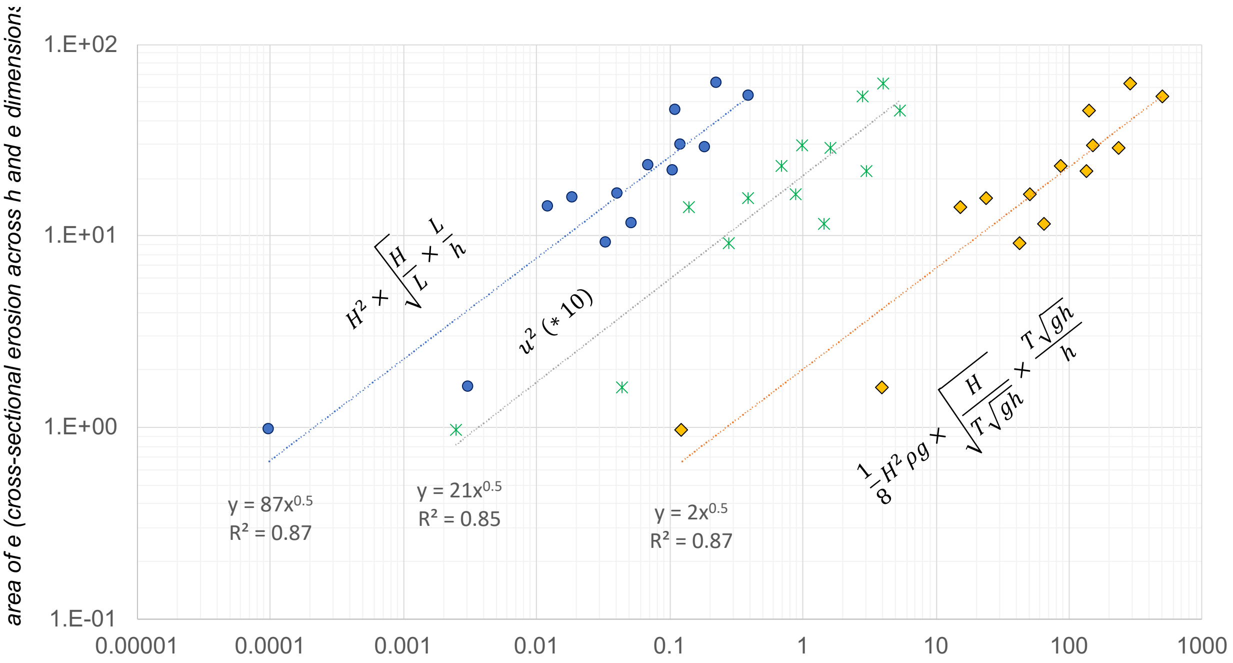

Fig. S6. Full datasets for *e* frequency distribution. In this graph, the data is not restricted to magnitudes greater than inflection point, i.e. at the mean of distribution, as in Fig. 2 in the main text. For H = 1 and H = 4, the power law extends across the entire domain and the inflection point lay below our detection limits.

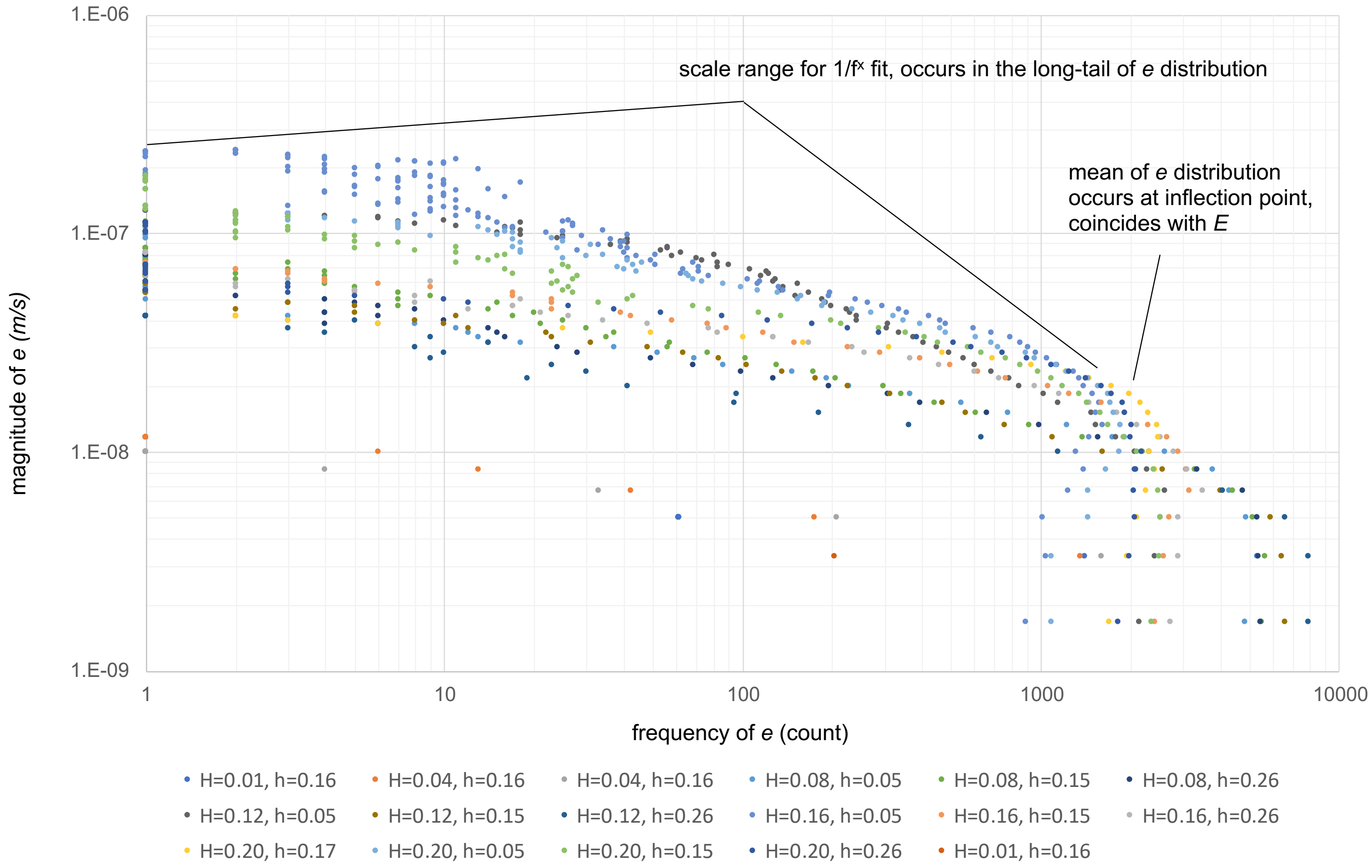

Fig. S7. Datasets for e frequency distributions, grouped by wave height H. Unique exponents and r^2 are shown, for each of the cases that contributed to the averaged $e = 1/f^{0.25}$. The average exponent across all fits was $f = -0.25$. However, each individual fit ranged from -0.11 to -0.34, with the lowest values corresponding to $H = 20$ with $h_* = 0.26$ and the highest to $H = 12$ with $h_* = 0.05$. The slope of the power function fit steepened when H^2/h_* increased. This result highlights the fact that the shape of the e erosion changes in response to wave form. The e distribution is steeper in slope (deeper holes) with plunging waves, and shallower with surging waves. Accordingly, one could solve the $E \propto e * h$ side of Eq. 1 for each of these cases separately, finding that H scales slightly differently in each case; however, for the full Eq. 1, the inclusion of h_* adequately accounts for this variation.

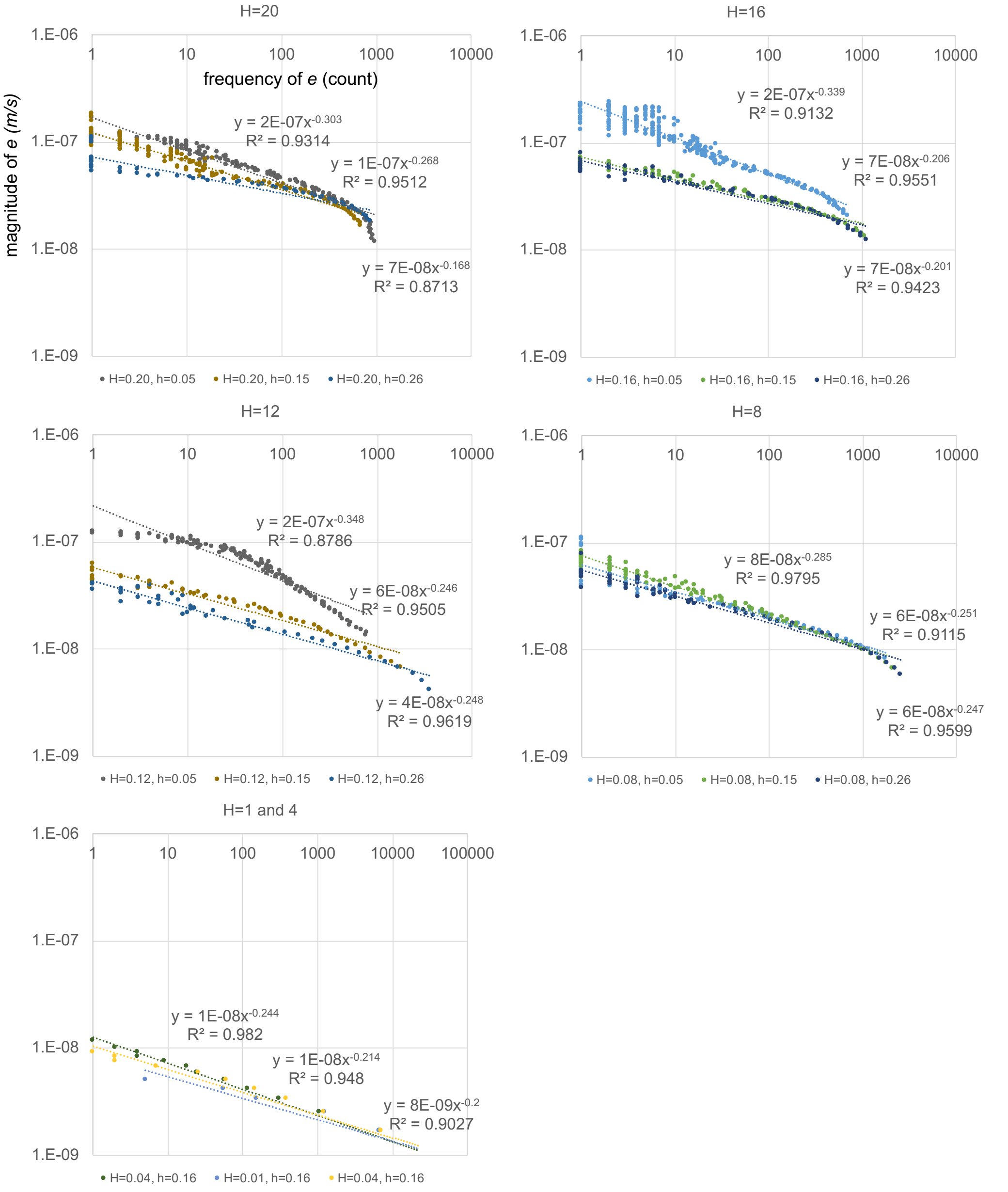

Fig. S8. For *e* individual erosion depths that are greater than the mean value (aka *E*), the distribution generally follows the power law relationship described in the text, across all H/h_* combinations. Values are ranked by magnitude on the x axis.

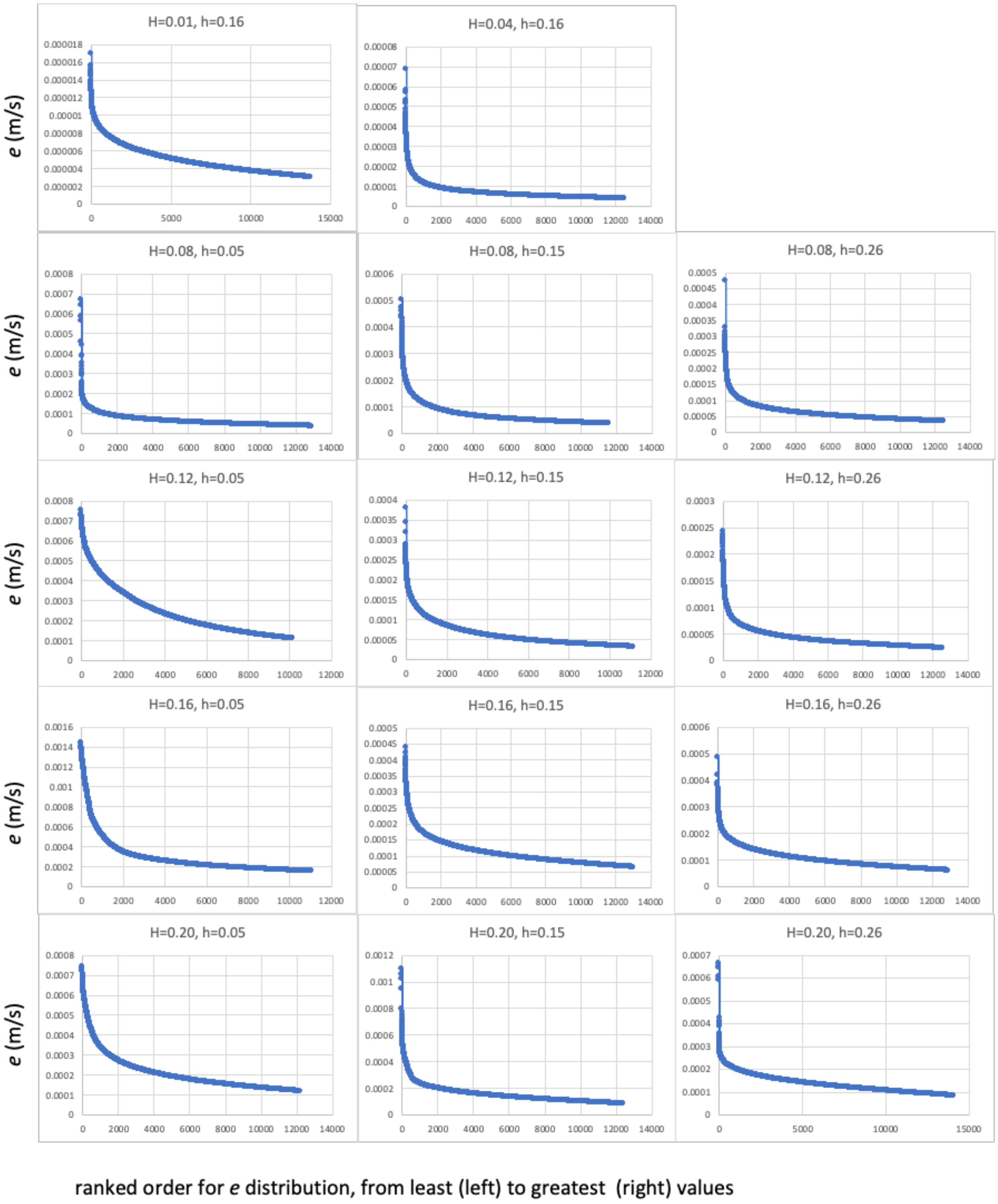

Fig. S9. For e individual erosion depths that are less than the mean value (aka E), the distribution generally follows the linear relationship described in the text, across all H/h_* combinations. Values are ranked by magnitude on the x axis.

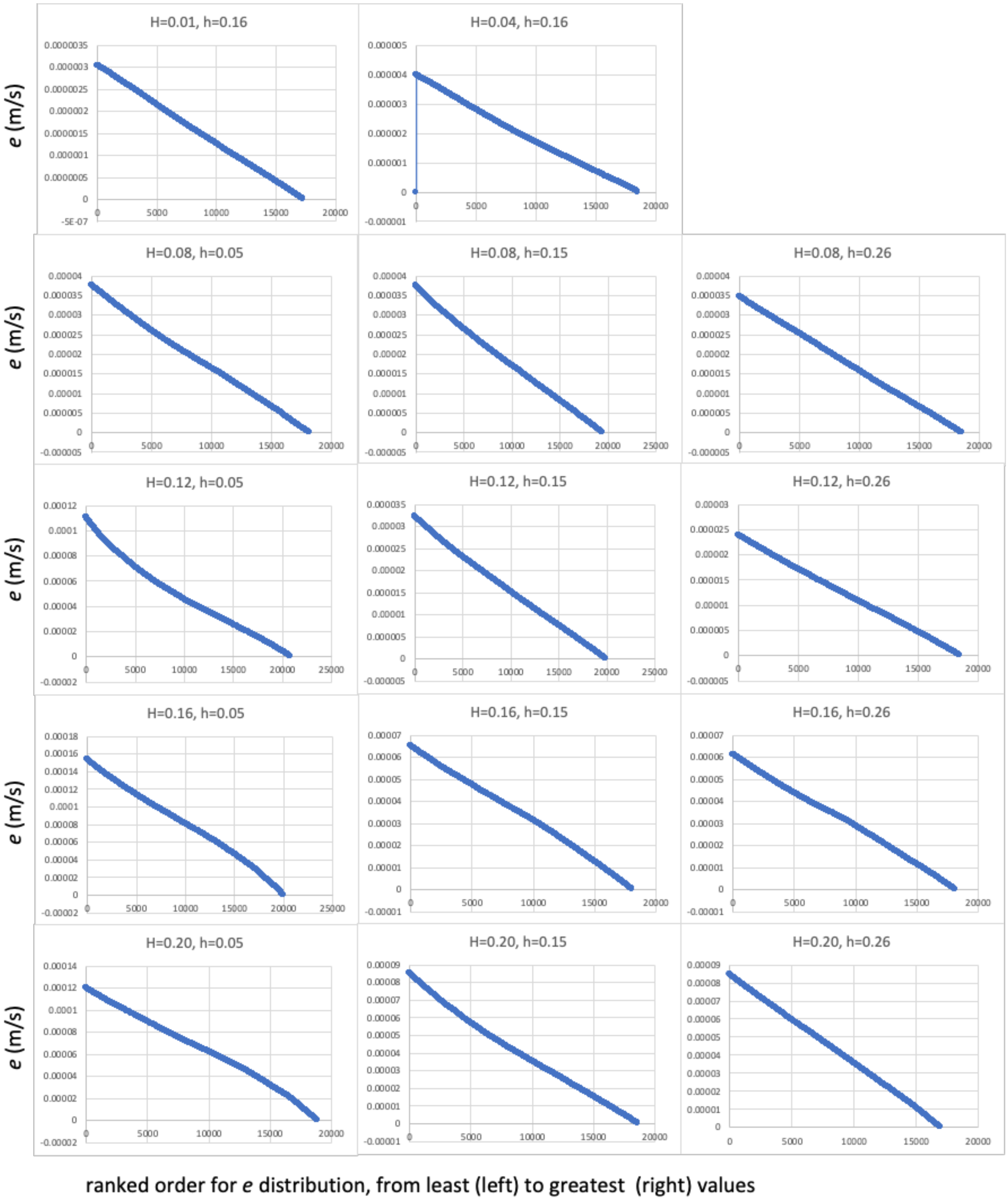

Movie S1. Waves intercepting the wetland edge samples during the laboratory portion of the study. The wetland edge samples were 0.6 x 0.3 m (horizontal footprint) x 0.15 m (vertical) in size, and were placed into a 22.9 x 36.6 x 1.2 m wave basin. (See online at *Science Advances*).

Supplementary Equations and Text

Derivations and Dimensionality of Equations

Dimensionality for Eq. 1

The units cancel out for Eq. 1 from the main text for $\zeta = 0.5$, making the relation dimensionless if stacked:

$$E \propto \left(H^2 \times \sqrt{\frac{H}{L}} \times \frac{L}{h_*}\right)^{\zeta}$$

$$\downarrow$$

$$m \propto \left(m^2 \times \sqrt{\frac{m}{m}} \times \frac{m}{m}\right)^{0.5}$$

$$\downarrow$$

$$1{:}\ 1$$

OR

$$\frac{E}{\left(H^2 \times \sqrt{\frac{H}{L}} \times \frac{L}{h_*}\right)^{\zeta}}$$

It should be pointed out that for the lab waves (from Fig. 1 in main text), we know that $\zeta = 0.5$. This exponent describes the conversion from a two-dimensional sine wave into a one-dimensional lateral erosion rate E. It also should be pointed out that both sides can also be presented in per-unit time, or the duration over which we allow this wave-to-erosion interaction to occur. So, we could express E as m/s instead of m, and implement the same for the entirety of the right-hand side.

Derivation of Eq. 2, Dimensionality, and Extension to Deepwater

Eq. 2 can be derived from Eq. 1, using $\zeta = 0.5$:

$$E \propto \left(H^2 \times \sqrt{\frac{H}{L}} \times \frac{L}{h_*} \right)^{0.5}$$

To do this, we first square both sides, then we insert the common terms for wave energy density $\frac{1}{8}\rho g H^2$ in place of H^2, and the shallow water wave period $T\sqrt{gh}$ in place of L. Next, to the left hand side, we add the bulk density of the sediment φ, and gravitational acceleration g when it moves:

↓

$$E^2 \varphi g \;=\; \frac{1}{8}\rho g H^2 \times \sqrt{\frac{H}{(T\sqrt{gh})}} \times \frac{T\sqrt{gh}}{h_*} \qquad \text{for shallow water}$$

↓

We then offset units and move the terms around to obtain Eq. 2:

$$E^2 \varphi g \;=\; H^{2.5} \times T^{0.5} \times \frac{\rho g^{1.25}}{8 h_*{}^{0.75}} \qquad \text{for shallow water}$$

The units can be cancelled out for Eq. 2 from the main text, also making its respective relationship dimensionless. Alternately, in order to express the two sides of each equation in terms of work (kilograms meters^2 seconds^{-2}), units can be selectively canceled out.

↓

$$m^2 * (kg/m^3) * (m/s^2) \;= m^{2.5} \;\times\; s^{0.5} \;\times\; \frac{(kg/m^3)*(m/s^2)^{1.25}}{m^{0.75}}$$

↓

$$m^2 * (kg/m^3) * (m/s^2) \;= m^{2.5} \;\times\; s^{0.5} \;\times\; \frac{(kg/m^3) * m^{0.5}}{s^{2.5}}$$

Cancelling and moving some terms around to make it more obviously symmetric on the two sides of the equation yields:

$$\downarrow$$

$$m^2 * (kg/m^3) * (m/s^2) = m^2 \times (kg/m^3) \times (m/s^2)$$

$$\downarrow$$

1 : 1

A deepwater version of Eq. 2 can alternately be created from Eq. 1, again using ζ = 0.5:

$$E \propto \left(H^2 \times \sqrt{\frac{H}{L}} \times \frac{L}{h_*} \right)^{0.5}$$

To do this, we similarly first square both sides, then we insert the common terms for wave energy density $\frac{1}{8}\rho g H^2$ in place of H^2, but now use the deepwater wave period $\frac{g}{2\pi}T^2$ in place of L. Next, to the left hand side, we add the bulk density of the sediment φ, and gravitational acceleration g when it moves:

$$\downarrow$$

$$E^2 \varphi g = \frac{1}{8}\rho g H^2 \times \sqrt{\frac{H}{\frac{g}{2\pi}T^2}} \times \frac{\frac{g}{2\pi}T^2}{h_*} \quad \text{for deep water}$$

$$\downarrow$$

We then offset units and move the terms around to obtain:

$$E^2 \varphi g = H^{2.5} \times T \times \frac{\rho g^{1.5}}{20 h_*} \quad \text{for deep water}$$

And the units can be similarly cancelled out, making its respective relationship dimensionless, or expressed in terms of work as mentioned above.

$$\downarrow$$

$$m^2 * (kg/m^3) * (m/s^2) \; = \; m^{2.5} \times s \times \frac{(kg/m^3)*(m/s^2)^{\;1.5}}{m}$$

$$\downarrow$$

Cancelling and moving some terms around to make it more obviously symmetric on the two sides of the equation yields:

$$\downarrow$$

$$m^2 * (kg/m^3) * (m/s^2) \; = m^2 \; \times (kg/m^3) \; \times \; (m/s^2)$$

1 : 1

The dimensionality, unit cancellation, and symmetry can be made far more elegant – and more obvious and easy to do - for both shallow water and deepwater relations, if instead of starting with Eq. 2 from the main text, one starts with the equation described in the subsection below entitled *Expanded Discussion…* The same basic procedure then follows of first squaring both sides, inserting the common terms for wave energy density, wave period, bulk density of the sediment and its gravitational acceleration.

Expanded Discussion: Why increasing the wave height results in greater erosion efficiency, but increasing the wavelength reduces it

The mechanics implicit in Eq. 1 appear more balanced if we square both sides, redistribute $\sqrt{H/L}$, and introduce a time duration t to both denominators:

$$\frac{E^2}{t\sqrt{H}} \propto \frac{H^2}{t\sqrt{L}} * \left(\frac{L}{h_*}\right)$$

The potential for a two-dimensional area of erosion E^2 (left hand side) is now divided by a reduced version of H in the denominator, which is to say that it is constrained along a reduced vertical dimension h_y. Similarly, the wave energy H^2 (right hand side) is constrained across a reduced horizontal dimension of the passing L. This formulation further suggests that the potential erosional area and wave energy are more-densely concentrated in specific segments of the wave form, across time.

As the wave height H independently increases, the erosional area numerator proportionally increases. However, the amount of time that the wave form intersects a discrete h_y vertical position on the edge decreases, on average, at the rate of its product with the square root of the wave height in the vertical dimension, $t\sqrt{H}$. This change in the time expended per differential unit of vertical edge occurs, because in order for a sine wave cycle to cover an increase in H across the entirety of L, given the same total time elapsed, the linear velocity along the sine wave must increase.

The rate of the linear velocity increase in the h dimension is directly proportional to the increase in H and is constant along the wave form, given a constant angular velocity (i.e., L is unchanged). However, the absolute magnitude of the increase along the wave form in the H dimension is inversely proportional to the distance from h_*; this is due to the nature of the sine function. In other words, the wave now spends considerably less absolute time intersecting the edge at its node h_* as compared to its crest or trough $h_{\pm y-limit}$.

If we multiply this time-spent per discrete h_y by the unchanged linear velocities in the E dimension (when L is unaltered), then the distribution of e is flattened relative to the h vertical dimension. Given constant linear and angular velocity in the horizontal dimension, the erosional face shallows in slope as H alone increases. Indeed, the rate of change in the perpendicular dimension (i.e., L), is different from that in the H (or, h) dimension, i.e., $e_y \propto 1/{h_y}^{0.25}$. The erosional shape is relative to the slope of the e distribution across H.

It follows that as the wavelength L alone independently increases (without altering H or the time duration t), the erosion decreases on a per increment of time basis at the rate of the square root of the increasing wavelength, $t\sqrt{L}$. However, the converse occurs on a per wave basis where the amount of time that the wave form intersects a discrete point of the edge increases.

The time units on either side of the above equation cancel, and so ultimately it can deliver a per wave formulation. Additionally, once H exceeds roughly double the water depth h_*, a wave breaks due to gravity. The water depth h_* can thus be seen as a modulator of the maximum realization of H^2 (i.e., H^2/h_*) or as a term in the inverse relative water depth (i.e., L/h_*). It accounts for variation in eroding edge heights and wave height-to-water depth effects, which also greatly affect the erosion *(8)*. Finally, as a general physical rule due to gravity and wave-

breaking processes, h_* or L cannot be infinitesimally small without H being similar in magnitude.

Our description of these wave-to-erosion mechanics should be considered valid only for erosion surfaces composed of consolidated sediments. Consolidated or cohesive sediments are defined herein as those sufficient to maintain vertical and horizontal integrity against gravity or buoyant forces, as long as the peds are stacked immediately on top of one another. Non-wetland and rocky cliffs likely function similarly to wetland edges when they are under wave attack. However, because these types of coastlines lie well above the water level for much longer time periods, gravity-induced mass failure and precipitation-induced erosion likely predominate *(18)* and affect the scaling of erosion.

See main text for the cited references.
